# Genetic and morphological variation in sexual and asexual parasitoids of the genus *Lysiphlebus* – an apparent link between wing shape and reproductive mode

**DOI:** 10.1186/s12862-015-0293-5

**Published:** 2015-02-04

**Authors:** Andjeljko Petrović, Milana Mitrović, Ana Ivanović, Vladimir Žikić, Nickolas G Kavallieratos, Petr Starý, Ana Mitrovski Bogdanović, Željko Tomanović, Christoph Vorburger

**Affiliations:** Institute of Zoology, Faculty of Biology, University of Belgrade, Studentski trg 16, 11000 Belgrade, Serbia; Department of Plant Pests, Institute for Plant Protection and Environment, Banatska 33, Zemun, 11080 Serbia; Department of Biology and Ecology, Faculty of Sciences and Mathematics, University of Niš, Višegradska 33, 18000 Niš, Serbia; Laboratory of Agricultural Entomology, Department of Entomology and Agricultural Zoology, Benaki Phytopathological Institute, 8 Stefanou Delta str., 14561 Kifissia, Attica Greece; Laboratory of Aphidology, Institute of Entomology, Biology Centre, Academy of Sciences of the Czech Republic, Branišovská 31, 37005 České Budějovice, Czech Republic; Institute of Biology and Ecology, Faculty of Science, University of Kragujevac, Radoja Domanovića 12, 34000 Kragujevac, Serbia; Institute of Integrative Biology, ETH Zürich, Switzerland, and EAWAG, Swiss Federal Institute of Aquatic Science and Technology, Überlandstrasse 133, 8600 Dübendorf, Switzerland; Laboratory of Agricultural Zoology and Entomology, Department of Crop Science, Agricultural University of Athens, 75 Iera Odos str., 11855 Athens, Attica Greece

**Keywords:** Parasitoid wasps, Wing shape, Reproductive mode, *COI*

## Abstract

**Background:**

Morphological divergence often increases with phylogenetic distance, thus making morphology taxonomically informative. However, transitions to asexual reproduction may complicate this relationship because asexual lineages capture and freeze parts of the phenotypic variation of the sexual populations from which they derive. Parasitoid wasps belonging to the genus *Lysiphlebus* Foerster (Hymenoptera: Braconidae: Aphidiinae) are composed of over 20 species that exploit over a hundred species of aphid hosts, including many important agricultural pests. Within *Lysiphlebus,* two genetically and morphologically well-defined species groups are recognised: the “*fabarum*” and the “*testaceipes*” groups. Yet within each group, sexual as well as asexual lineages occur, and in *L. fabarum* different morphs of unknown origin and status have been recognised. In this study, we selected a broad sample of specimens from the genus *Lysiphlebus* to explore the relationship between genetic divergence, reproductive mode and morphological variation in wing size and shape (quantified by geometric morphometrics).

**Results:**

The analyses of mitochondrial and nuclear gene sequences revealed a clear separation between the “*testaceipes*” and “*fabarum*” groups of *Lysiphlebus*, as well as three well-defined phylogenetic lineages within the “*fabarum*” species group and two lineages within the “*testaceipes*” group. Divergence in wing shape was concordant with the deep split between the “*testaceipes”* and “*fabarum”* species groups, but within groups no clear association between genetic divergence and wing shape variation was observed. On the other hand, we found significant and consistent differences in the shape of the wing between sexual and asexual lineages, even when they were closely related.

**Conclusions:**

Mapping wing shape data onto an independently derived molecular phylogeny of *Lysiphlebus* revealed an association between genetic and morphological divergence only for the deepest phylogenetic split. In more recently diverged taxa, much of the variation in wing shape was explained by differences between sexual and asexual lineages, suggesting a mechanistic link between wing shape and reproductive mode in these parasitoid wasps.

**Electronic supplementary material:**

The online version of this article (doi:10.1186/s12862-015-0293-5) contains supplementary material, which is available to authorized users.

## Background

The morphological diversity of the living world, including the variation in size and shape, is generally assumed to be adaptive and shaped by natural selection. However, numerous internal factors, such as shared developmental systems inherited from common ancestors as well as structural and functional constraints, can restrict the response to selection [1-3]. An independently derived phylogeny is required to disentangle the effects of these influences on morphological evolution [4,5]. Although numerous studies have investigated morphological evolution in a phylogenetic context in various groups [6-9], this approach is still relatively rare in the studies of morphological variation in parasitoid wasps, even though they represent a very interesting biological model. Parasitoids are hyperdiverse (together with their hosts and host plants representing half of the world’s biodiversity [10,11]) and they possess complex life cycles and morphologies shaped by intimate host-parasitoid interactions. The frequent occurrence of both sexual and asexual reproduction poses a particular challenge because asexual lineages can freeze and amplify particular portions of morphological variation.

We addressed these issues in aphid parasitoids belonging to the genus *Lysiphlebus* Foerster (Braconidae: Aphidiinae). They are solitary koinobiont parasitoids, meaning that a single parasitoid larva develops per aphid host and allows the host to continue its growth and development while feeding upon it. *Lysiphlebus* exploits over a hundred species of mostly small aphids, including many important pests (e.g., soybean aphid, *Aphis glycines* Matsumura; black bean aphid, *A. fabae* Scopoli; cotton aphid, *A. gossypii* Glover) [12,13].

**Figure 1:**
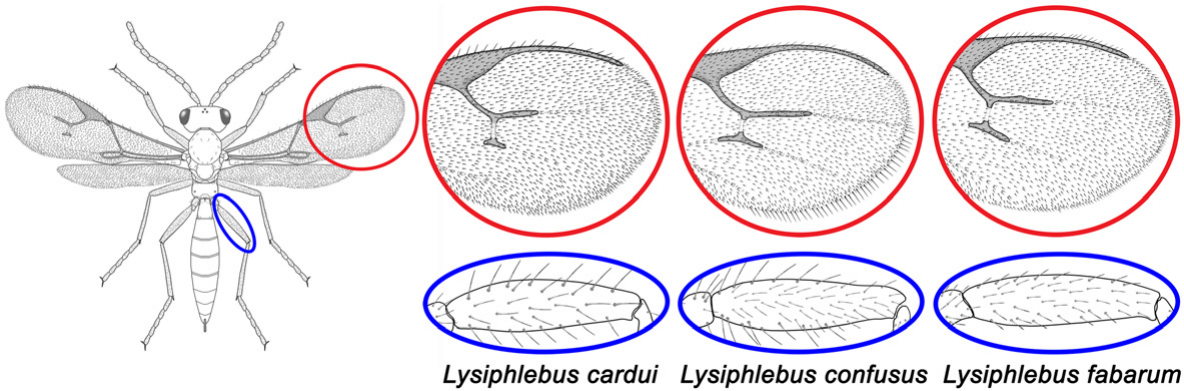
**Morphological differences among the** ***fabarum*** **,** ***cardui*** **and** ***confusus*** **morphs of** ***Lysiphlebus fabarum*** **.** Red circles – anterior part of forewing, showing setae on the distal margin; blue ellipses – femurs of the hind legs.

Two species groups are recognised within the genus *Lysiphlebus* based on wing morphology [14] and molecular evidence [15]: the “*fabarum*” group and the “*testaceipes*” group. Taxa with sexual and asexual reproduction are present within both species groups, and they show some divergence in early life history traits and morphological traits [16], including wing shape [17]. For example, the species *Lysiphlebus hirticornis* Mackauer and *L. melandriicola* Starý (“*fabarum*” group), as well as *L. testaceipes* (Cresson) (“*testaceipes*” group), such as most Hymenoptera, reproduce by arrhenotoky (sexual reproduction) [18,19], in which fertilised eggs develop as females and unfertilised eggs develop as haploid males. By contrast, a recently described species of the “*testaceipes*” group, *L. orientalis* Starý & Rakhshani, reproduces exclusively by thelytoky (asexual reproduction) [14,15], that is by producing diploid females without fertilisation. *Lysiphlebus balcanicus* Starý, an endemic species of southeastern Europe also belonging to the “*testaceipes*” group, appears to be thelytokous as well, because only female specimens were encountered in samples collected over a long period of time [12,20,21]. Within the “*fabarum*” species group, three of the described species, namely *L. fabarum* (Marshall), *L. cardui* (Marshall) and *L. confusus* (Tremblay and Eady), occur in both sexual (arrhenotokous) and asexual (thelytokous) populations [22-24]. These three species were described based on morphological differences in the length and orientation of setae on the hind legs and on the distal margin of the wings, by which they are clearly distinguishable [24] (Figure 1). However, different genetic studies showed no or little support for the species status of these three taxa [22,24,25]. We will therefore refer to them as the *fabarum*, *confusus* and *cardui* morphs [24].

The polygenic basis of wing shape and its adaptive significance (flight, host finding), combined with the relative simplicity of a two-dimensional structure that allows a precise recording and analysis of the shape using geometric morphometrics, makes wing shape a very useful trait in the study of morphological evolution [7,26-28]. Here, we explored the molecular variation (based on nuclear and mitochondrial genes) and morphological variation (based on wing shape) to analyse possible factors, including reproductive mode, that are associated with the evolution of wing shape in aphid parasitoids from the genus *Lysiphlebus*. Specifically, we addressed the following questions: i) Is genetic differentiation accompanied by parallel changes in the shape of the wing? ii) Are parasitoid wasps assigned to different morphs and/or with different reproductive modes also divergent in their wing shape?

## Results

### Genetic diversity

**Figure 2:**
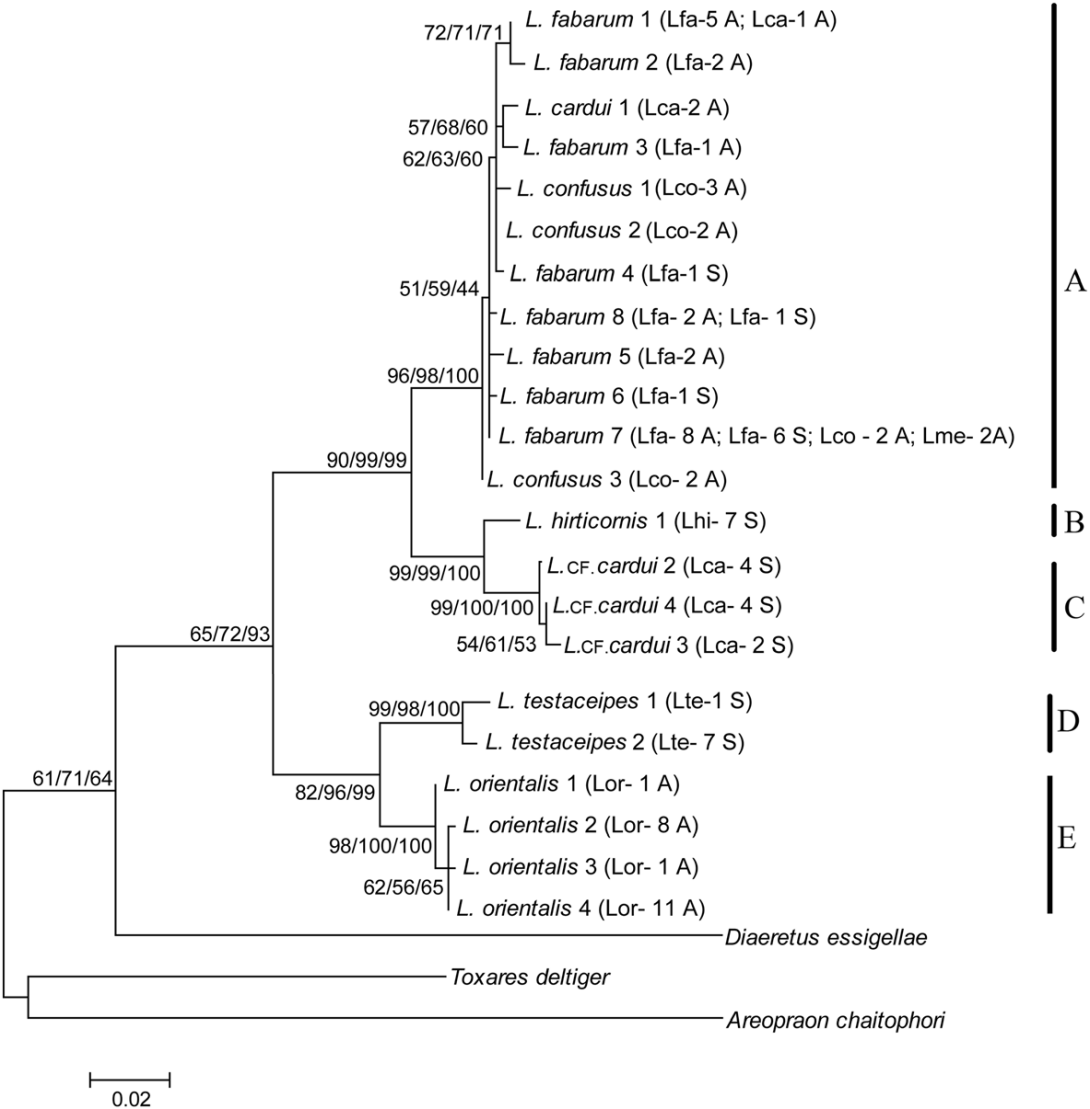
**Maximum Likelihood phylogenetic tree of** ***Lysiphlebus*** **haplotypes obtained from** ***COI*** **sequences.** Numbers above/below the branches represent the bootstrap values (only values above 50% are shown) in the following order: Maximum Likelihood/Maximum Parsimony/Neighbour Joining. The scale bar indicates substitutions per site. Species/morphs, number of specimens and mode of reproduction are indicated in parentheses next to haplotypes: Lfa – *L. fabarum* morph, Lca – *L. cardui* morph*,* Lco *– L. confusus* morph*,* Lme – *L. melandriicola*, Lhi *– L. hirticornis,* Lte *– L. testaceipes,* Lor *– L. orientalis*; A – asexual mode of reproduction, S - sexual mode of reproduction. Vertical bars marked **A**, **B**, **C**, **D** and **E** represent phylogenetic clades used in morphometric analyses (**A** – *L. fabarum* asexual and sexual, *L. confusus* asexual, *L. cardui* asexual, *L. melandricola* asexual; **B** – *L. hirticornis*; **C** – *L. cardui* sexual; **D** – *L. testaceipes*; **E** – *L. orientalis*). For more haplotype information, see Additional file 1.

All sequences of the amplified cytochrome oxidase subunit I gene (*COI*) of *Lysiphlebus* specimens were indel-free. The total alignment of 628-bp long mitochondrial sequences contained 83 variable sites, of which 70 were parsimony informative. The topologies of Maximum Parsimony (MP), Maximum Likelihood (ML) and Neighbour-Joining (NJ) trees were highly congruent. For this reason, only the ML tree is shown in Figure 2, although the bootstrap support obtained by all three methods of phylogenetic analyses is indicated in the figure.

Analyses of mitochondrial sequences supported the separation of the genus into the “*fabarum*” (clades A, B, C; Figure 2) and the “*testaceipes*” (clades D, E; Figure 2) species groups, with a mean genetic distance of 7.6% between the groups.

Within the “*testaceipes*” group, *L. orientalis* (clade E) and *L. testaceipes* (clade D) split as separate taxa, with a mean genetic distance of 3.7%. *L. testaceipes* consisted of two distinct haplotypes with 1% genetic divergence, whereas four haplotypes were recorded in *L. orientalis* (0.3% mean genetic distance).

Based on the *COI* gene analysis, the “*fabarum*” group was composed of 16 distinct haplotypes that are clustered into three well-supported phylogenetic lineages (A, B, C in Figure 2). Overall, the mean genetic distance among specimens within the “*fabarum*” group was 2.2%.

Most of the haplotypes (12) were grouped into phylogenetic clade A. Within this clade, the tree topology had poor statistical support, and the highest genetic distance between two haplotypes was 1.1% (mean distance was 0.6%). Two haplotypes were found in both asexual and sexual parasitoids (*L. fabarum* 7 and *L. fabarum* 8), two were found only in sexual parasitoids, and eight were found only in parasitoids with asexual reproduction (Figure 2, Additional file 1).

Associations of *COI* haplotypes with the three morphs of *L. fabarum* were inconsistent. Most haplotypes were associated with one morph, but *L. fabarum* 1 was associated with the *fabarum* and *cardui* morphs, whereas the most common haplotype, *L. fabarum* 7, was associated with the *fabarum* and *confusus* morphs, but also with *L. melandriicola*.

Clade B was represented only with one haplotype, which is associated with all *L. hirticornis* specimens. *Lysiphlebus hirticornis* is clearly separated from clade A with a mean genetic distance of 4.1%.

All *cardui* morphs with sexual reproduction are grouped in phylogenetic clade C and separated from all other species/morphs. There is a genetic distance of 4.7% between these morphs and the same morphs with an asexual mode of reproduction that are all grouped in clade A. This provides strong evidence that these sexual parasitoids represent a yet undescribed species. The phylogenetic trees showed that the closest relatives of sexual *cardui* morphs in our material are the specimens belonging to *L. hirticornis* (2% divergence from *L. hirticornis* 1 haplotype).

The analysis of nuclear sequences (*28S D2* gene) showed a well-supported split (100/99/99, Additional file 2) between outgroups and *Lysiphlebus* parasitoids, with a sequence divergence ranging from 11.2 to 26.7%.

All *Lysiphlebus* specimens clustered into two groups corresponding to the “*fabarum*” group (*L. fabarum* 1–4, *L. cardui* 1–2, *L. confusus*, and *L. hirticornis* 1 haplotypes) and the “*testaceipes*” group (*L. orientalis* 1, 2 and *L. testaceipes* 1 haplotype) (Additional file 2). The mean genetic divergence of the nuclear *28S D2* sequences between the “*fabarum*” and “*testaceipes*” species group was 3.1%.

Within the two species groups, the phylogenetic tree based on *28S D2* was poorly resolved due to insufficient sequence variation (average difference within species groups was only 0.1%).

### Morphological differences

The five distinct *Lysiphlebus* phylogenetic lineages based on *COI* (A, B, C, D, and E; Figure 2) significantly differed in wing size (ANOVA, F_4,187_ = 28.54, P < 0.001) and in wing shape (MANOVA, Wilks' Lambda = 0.412, F_80, 645.44_ = 2.04, P < 0.001). The comparisons of wing shape expressed as Procrustes distances revealed significant differences in all pairwise comparisons (Table 1, right to the diagonal), whereas differences in wing size were less pronounced (Table 1, below diagonal).

**Table 1:** **Mean and standard deviation of wing size (CS) per phylogenetic lineage**

	**n**	**mean size ± StDev**	**A**	**B**	**C**	**D**	**E**
A	112	1336.36 ± 102.35		**0.038**	**0.030**	**0.120**	**0.163**
B	21	1073.80 ± 61.51	**259.72**		**0.039**	**0.138**	**0.181**
C	15	1252.34 ± 118.24	87.02	**178.54**		**0.141**	**0.185**
D	15	1171.66 ± 127.08	**164.70**	95.02	12.55		**0.050**
E	29	1276.62 ± 64.68	59.74	**199.98**	50.15	**37.60**	

Differences in wing size between lineages (absolute values of differences in CS) are given below diagonal. The differences in wing shape expressed by Procrustes distances are given to the right of the diagonal. Results significant at P < 0.05 are shown in boldface type.

**Figure 3:**
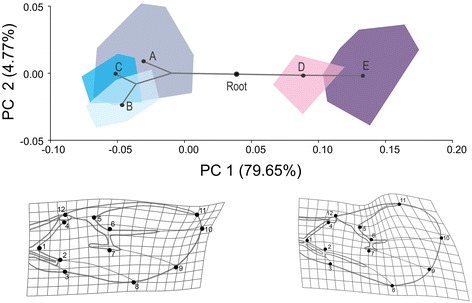
**The wing shape morphospace defined by the first two axes obtained from principal component analysis and the superimposed** ***Lysiphlebus*** **phylogeny.** The shape changes along the first axis are presented as deformation grids. Letters (A, B, C, D and E) represent phylogenetic clades based on the *COI* gene.

The principal component analysis (Figure 3) showed that clades A, B, and C clearly differ from clades D and E along the first principal axis, which describes almost 80% of the total variance in wing shape. The main shape changes that separate the ABC (“*fabarum*” group) and DE clades (“*testaceipes*” group) are related to changes in the stigma, the length of the R1 vein (landmarks 5, 11, 12) and the width of the wing’s distal part. Compared to the “*fabarum*” group (ABC), “*testaceipes*” group parasitoids (DE) have a wider distal part of the wing (landmarks 6, 7, 8, 9, 10 and 11), a shorter R1 vein and a wider stigma.

The permutation test against the null hypothesis of no phylogenetic signal revealed no evidence of a phylogenetic signal in wing size (tree length = 0.0231, P = 0.674) but did reveal a significant phylogenetic signal in wing shape (tree length = 0.0094, P = 0.037). The superimposition of the phylogenetic tree in the morphospace of the first two PC axes shows that concordance in divergence between genetic and morphological variation is due to the divergence between the two main clades (Figure 3).

Separate PCAs of wing shape variation within the “*fabarum*” (ABC) and “*testaceipes*” (DE) groups reveal that the divergence in wing shape is largely associated with reproductive mode. Within clade A, four subgroups can be distinguished *a priori,* based on the combination of morph (see Figure 1) and reproductive mode: A1 – *L. cardui*/asexual, A2 – *L. confusus*/asexual, A3 – *L. fabarum*/asexual and A4 – *L. fabarum*/sexual). Clades B (*L. hirticornis*/sexual) and C (*L. cardui*/sexual) are uniform with respect to these criteria.

**Figure 4:**
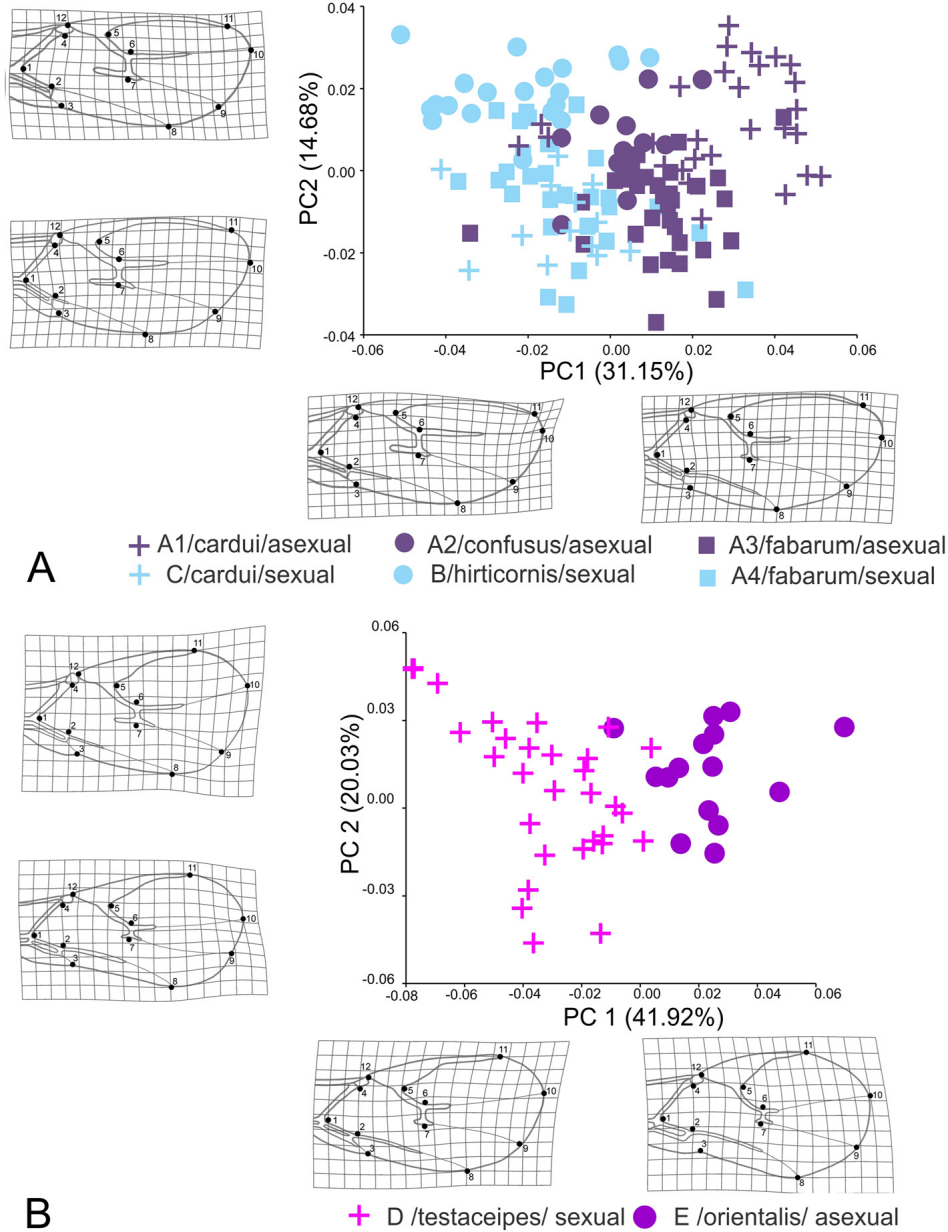
**Position of individuals representing** ***Lysiphlebus*** **“** ***fabarum*** **” and “** ***testaceipes*** **” species groups over the first and second Principal Component (PC) axis. A** - Principal component analysis for “*fabarum*” group (clades ABC). Note that lineage A consists of four groups (A1 – *L. cardui*/asexual, A2 – *L. confusus*/asexual, A3 – *L. fabarum*/asexual and A4 – *L. fabarum*/sexual). **B** – Principal component analysis for “*testaceipes*” group (clades DE). The observed morphological differentiation is summarised by deformation grids.

In the morphospace defined by the first two principal components that describe over 45% of the total variation in wing shape, sexual and asexual lineages within the “*fabarum*” group clearly separate along the first axis (Figure 4A). Sexual lineages had longer and narrower forewings; particularly the apical part of the wing (area between stigma and first radial nerve) is narrower in the sexual lineages compared with asexual lineages. The analysis of wing shape variation in the “*testaceipes*” group (DE) showed that *L. orientalis* (E) and *L. testaceipes* (D) are clearly separated in the morphospace defined by the first two principal axes that describe almost 62% of total variance in wing shape. Notably, the differences between the sexual and the asexual wasps parallel those observed in the “*fabarum*” group. The wings of the asexual species *L. orientalis* are characterised by a wider distal part and a shorter R1 vein in comparison with the sexual species *L. testaceipes* (Figure 4B).

**Figure 5:**
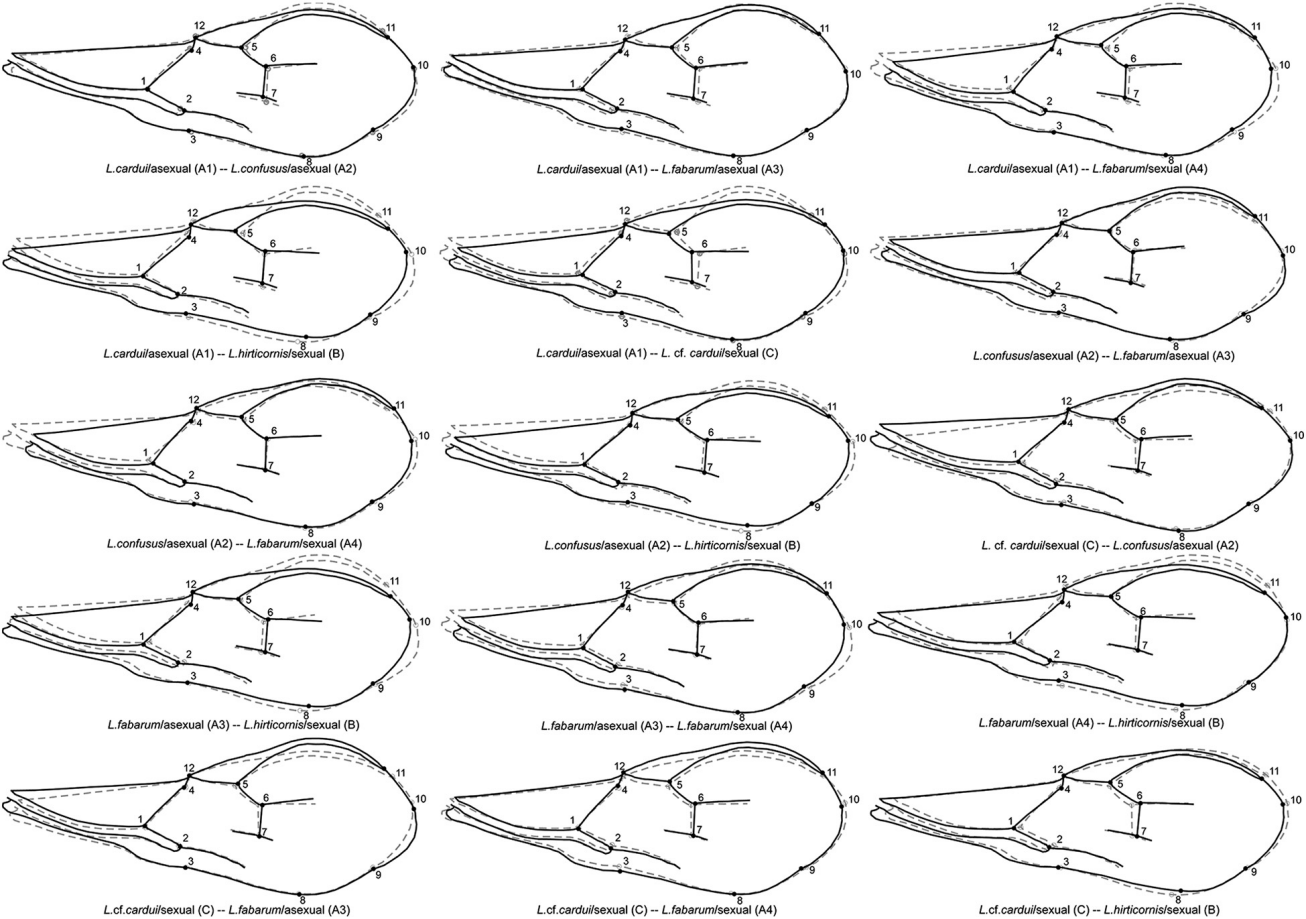
**Illustration of wing shape differences between** ***a priori*** **groups of** ***Lysiphlebus*** **parasitoids.** The shape changes are shown as the difference between the average shape of the first group (grey dashed lines and grey circles) and the average shape of the second group (black outlines and solid circles). All changes are enlarged 1.5 times.

Pairwise comparisons within the “*fabarum*” group revealed that sexual *L. fabarum (*A4) have significantly smaller wings than all three asexual groups within clade A. Two sexual lineages, *L. hirticornis* (clade B) and *L. cardui* (clade C), have smaller wing size compared with all other groups except for sexual *L. fabarum* (A4) and sexual *L. cardui* (clade C) (Table 2). All groups based on morph and/or reproductive mode showed significant divergence in wing shape (Table 2, Figure 5).

**Table 2:** **Differences in wing size (below diagonal) and wing shape (above diagonal) between** ***a priori*** **groups based on morph and/or reproductive mode**

	**n**	**Mean size ± StDev**	**A1**	**A2**	**A3**	**A4**	**B**	**C**
A1 *L. cardui*/asexual	29	1360.1 ± 110.6		**0.028**	**0.030**	**0.041**	**0.049**	**0.048**
A2 *L. confusus*/asexual	14	1396.3 ± 32.0	36.19		**0.024**	**0.027**	**0.033**	**0.031**
A3 *L. fabarum*/asexual	36	1347.1 ± 97.2	12.95	49.14		**0.032**	**0.045**	**0.031**
A4 *L. fabarum*/sexual	29	1273.8 ± 86.1	**86.3**	**122.49**	**73.35**		**0.035**	**0.028**
B *L. hirticornis*/sexual	20	1073.8 ± 61.5	**286.29**	**322.48**	**273.34**	**199.99**		**0.038**
C *L. cardui*/sexual	14	1252.3 ± 118.2	**107.75**	**143.94**	**94.8**	21.45	**178.54**	

Absolute values of differences in CS are given for wing size. The differences in wing shape expressed by Procrustes distances are given. Results significant at P < 0.05 are shown in boldface type.

Correct classification of individuals into the *a priori* groups was (values after cross-validation in parentheses): A1 – asexual *L. cardui* 97% (86%); A2 – asexual *L. confusus* 100% (93%); A3 – asexual *L. fabarum* 100% (89%); A4 – sexual *L. fabarum* 100 (62%); B – sexual *L. hirticornis* 100 (85%); and C – sexual *L. cardui* 100 (79%).

*L. testaceipes* (D) and *L. orientalis* (E) also differ clearly in wing size and wing shape (Procrustes distance is 0.051, P < 0.001). Correct classification of individuals was D – *L. testaceipes* 100% (98%) and E – *L. orientalis* 100% (100%).

The shape changes between pairs of *a priori* groups are illustrated in Figure 5. As previously described, the sexual and asexual lineages diverge in the shape of the distal part of the wing (described by landmarks 6, 7, 8, 9, 10 and 11), whereas different morphs often diverge in the shape of proximal area of the wing (described by landmarks 1, 2, 3, 4, 5 and 12).

**Figure 6:**
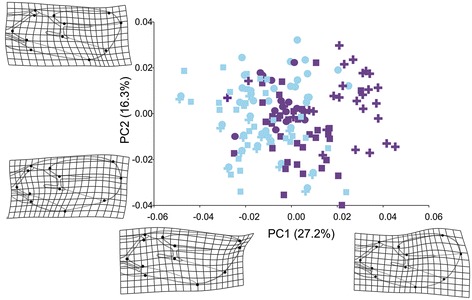
**The position of the individuals of** ***Lysiphlebus*** **“** ***fabarum*** **” species group over the first and second Principal Component (PC) axes after the correction for size.** Symbols and colour codes are as indicated in Figure 3. The observed morphological differentiation is summarised by deformation grids.

The multivariate regression of wing shape variables on log CS within the “*fabarum*” group was statistically significant (P < 0.0001): 10.3% of the total variation in wing shape could be explained by allometry. Within the “*testaceipes*” group, multivariate regression was not statistically significant (P = 0.099). To explore whether the observed shape changes within the “*fabarum*” group are due to divergence in size between groups and related allometric shape changes, we performed PCA on an allometry-free dataset (residuals obtained from multivariate regression of shape variables an log CS). Analysis of allometry-free shape variables revealed that the sexual and a sexual lineages differ in forewing shape regardless to differences in wing size between lineages (Figure 6).

## Discussion

By analysing the sequences of nuclear and mitochondrial genes we confirmed the existence of two previously acknowledged species groups (“*testaceipes*” and “*fabarum*”) within the genus *Lysiphlebus* [14]. In terms of taxonomic characterisation, the barcoding region of the *COI* gene has proven to be a suitable marker for species identification within the genus *Lysiphlebus* [15,24,25,29], whereas the more conservative nuclear *28S D2* gene appears to be informative only at the generic or species group level. An unexpected discovery was a distinct group of *COI* haplotypes (clade C, Figure 2) that comprised all *cardui* morphs (Figure 1) with a sexual mode of reproduction. It represents a yet undescribed species that deserves further taxonomic treatment.

Marked divergence in wing shape was found between the “*testaceipes*” and “*fabarum*” species groups. However, within these groups, especially within the “*fabarum*” group, there is no clear correspondence between the variation in wing shape and genetic divergence. Additionally, we did not find any concordance between wing shape and the three described morphs in *L. fabarum*.

A significant finding in our study is that reproductive mode (namely sexual vs. asexual reproduction) is associated with wing shape. Similar shape changes between the taxa with asexual and sexual modes of reproduction were recorded in the “*testaceipes*” as well as in the “*fabarum*” group. The relationship between wing shape and reproductive mode is particularly notable in the “*fabarum*” species group, in which most of the morphological variation could be related to the differences between the sexual and asexual taxa and morphs.

Taxa with sexual reproduction had smaller wings than the taxa with asexual reproduction, and there were significant, size-related allometric changes in the shape of the wing. However, divergence in the wing shape was not solely the result of allometry, because the sexual and asexual lineages differed in wing shape even when the size effect was removed (allometry-free data).

Sexual reproduction (arrhenotoky) is the ancestral and dominant mode of reproduction in Hymenoptera. Asexual reproduction (thelytokous parthenogenesis) is less frequent and often induced by heritable endosymbiotic bacteria [18,19], but apparently not in *Lysiphlebus*. Sandrock and Vorburger [30] showed that asexual reproduction in *L. fabarum* is genetically determined and inherited as a single-locus recessive trait. Asexual reproduction has the potential to spread in parasitoid populations because, very rarely, asexual (thelytokous) females produce fertile haploid males that may carry the thelytoky-inducing allele into sexual populations, a process referred to as ‘contagious parthenogenesis’ [31]. There are currently no data about the underlying processes of asexual reproduction in *L. orientalis*, but the simplest assumption would be that they are similar to those in *L. fabarum*, considering that thelytoky is otherwise very rare in the Aphidiinae and that rare males are also observed in natural and laboratory populations of *L. orientalis* [14]. In this study, the association of mitochondrial haplotypes with the mode of reproduction in the “*fabarum*” group was inconsistent, and two haplotypes were shared between the asexual and sexual wasps. These results are in concordance with the statement that the “*fabarum*” group is an evolutionary young sexual-asexual complex with incomplete genetic isolation between the reproductive modes [24].

The mechanistic basis of the relationship between wing shape and reproductive mode is currently unknown. There is a lack of information on the genetic basis of wing morphogenesis in parasitic wasps, but studies on *Drosophila melanogaster* show that numerous genes of small effect determine wing shape in flies [32]. Presuming that parasitoid and fly wing morphogenesis is not dramatically different, and considering that reproductive mode in *L. fabarum* is inherited as a single-locus trait [30], pleiotropy or co-inherence of the thelytoky-inducing allele and alleles affecting wing shape in the same linkage group may explain the observed link between reproductive mode and wing shape. This remains to be investigated.

## Conclusions

Deep genetic divergence of aphid parasitoids from the genus *Lysiphlebus* (the “*fabarum*” vs. “*testaceipes*” group) is accompanied by changes in wing shape. At a shorter timescale within the two species groups, there is no clear correspondence between morphological evolution and genetic divergence. However, we observed a clear association between reproductive mode and wing shape; in both species groups, similar differences exist between sexual and asexual wasps, explaining much of the variation in wing shape. Further studies are necessary to resolve the mechanisms underlying the apparent relationship between reproductive mode and wing shape in *Lysiphlebus* wasps.

## Methods

### Field sampling and determination of reproductive modes

*Lysiphlebus* parasitoids were sampled between 2006 and 2011 in the surroundings of Belgrade, Serbia, except for *L. testaceipes*, which was collected along the Mediterranean coast of Montenegro. In addition to those specimens, for molecular analyses we used samples from other geographical areas (Additional file 1). Plant samples infested with live and mummified aphids were collected in the field and placed into plastic containers covered with nylon mesh [33]. Aphid hosts were mainly from the genera *Aphis* and *Brachycaudus,* except for *Metopeurum fuscoviridae* Stroyan, which is parasitised by the monophagous parasitoid *L. hirticornis* (Additional file 1). Caged samples were held at 22.5°C until parasitoid emergence. Four collected species have known modes of reproduction: *L. hirticornis* – sexual; *L. melandriicola* – sexual; *L. testaceipes* – sexual; and *L. orientalis* – asexual. For the *fabarum*, *cardui* and *confusus* morphs of *L. fabarum* (see Figure 1), in which sexual and asexual lineages may occur, reproductive modes were mostly inferred from the field sex ratios. The complete absence of males was taken as an indicator of asexual reproduction, whereas samples containing males and females were treated as lineages with a sexual mode of reproduction. This approach is not applicable to samples with only a few individuals who are all female. To determine the reproductive modes of females from small samples (<5 individuals), we genotyped them at microsatellite locus *Lysi07* [34], which happens to be linked to reproductive mode in parasitoids of the *L. fabarum* group [30]. All females that were homozygous for allele 183 at microsatellite locus *Lysi07* were treated as asexual, whereas all others were treated as sexual [30].

In this study we analysed the following *Lysiphlebus* species/morphs: *L. fabarum* – sexual and asexual; *L. cardui* – asexual; *L. confusus* – asexual; *L. hirticornis* – sexual. We also analysed sexual lineages of *L.* cf. *cardui*, which is here recorded for the first time. Sexual specimens of *L. melandriicola* were used only for molecular analyses due to insufficient number of individuals available for analyses of the wing shape. All of the above belong to the “*fabarum*” group. From the “*testaceipes*” group, we used *L. testaceipes -* sexual and *L. orientalis* – asexual (Additional file 1). Only female specimens were included in all molecular and morphometric analyses.

### DNA extraction, PCR amplification and sequencing

To determine the genetic variation and phylogenetic relationships among the *Lysiphlebus* species/morphs, two molecular markers were chosen. The second expansion segment of the nuclear *28S rRNA* gene (28S D2) was amplified using the primer pair 28SD2f (5’-AGAGAGAGTTCAAGAGTACGTG-3’) [35] and 28SD2r (5’-TTGGTCCGTGTTTCAAGACGGG-3’) [36]. Additionally, the barcoding region of the mitochondrial cytochrome oxidase subunit I gene (*COI*) was amplified using the primers LCO1490 and HCO2198 [37]. Total nucleic acids were extracted from 89 *Lysiphlebus* specimens (Additional file 1) and three outgroup taxa (*Diaeretus essigellae* Starý & Zuparko 2002, *Areopraon chaitophori* Tomanović & Petrović 2009 and *Toxares deltiger* Haliday 1883) using a nondestructive TES method [38]. PCR reactions were performed in an Eppendorf Mastercycler® (Hamburg, Germany). Fragments of 28S D2 were amplified in a final volume of 20 μl, containing 1 μl of extracted DNA, 14.35 μl of H_2_0, 2 μl of High Yield Reaction Buffer A, 1.5 μl of MgCl_2_ (2.25 mM), 0.5 μl of dNTP (0.25 mM), 1 μl of each primer (0.5 μM) and 0.15 μl of KAPA*Taq* DNA polymerase (0.0375 U/μl) (Kapa Biosystems Inc., Boston, USA). The PCR protocol included initial denaturation at 95°C for 3 min, 30 cycles consisting of 30 s at 95°C, 30 s at 48°C, 2 min at 72°C, and the final extension at 72°C for 10 min. All *COI* products were amplified according to the protocol and cycling conditions described by Petrović et al. [15]. The PCR products were checked on 1% agarose gels and purified using the QIAquick PCR Purification Kit (QIAGEN, Valencia, USA) according to the manufacturer’s instructions. DNA sequencing was performed by Macrogen Inc. (Seoul, Korea).

### Phylogenetic analyses

Sequences of both genes were manually edited in FinchTV v.1.4.0 (Geospiza, Inc., Seattle, USA; http://www.geospiza.com) and aligned using the Clustal*W* program implemented in the MEGA 5.1 software package [39]. The same software was used to confirm the continuous open reading frame in the protein-coding *COI* gene to exclude the possibility of nuclear copies [40,41]. Kimura’s two-parameter method (K2P) of base substitution [42] was used to calculate an average genetic distance between the sequences.

Phylogenetic reconstruction was carried out using the methods of Maximum Likelihood (ML), Maximum Parsimony (MP) and Neighbour-Joining (NJ), all computed using the MEGA 5.1 software package. The MP tree was obtained using the Subtree-Pruning-Regrafting (SPR) algorithm [43]. The NJ phylogenetic tree was inferred using the K2P evolutionary distances [42]. For the reconstruction of the ML tree for 28S D2 sequences, we have identified the GTR + G model as the best-fitting model of sequence evolution based on the Bayesian Information Criterion (BIC) and the Akaike Information Criterion corrected (AICc) [43] as implemented in the software Modeltest 3.5 [44]. Based on the same criteria, the HKY + G model was identified as the best-fitting model for ML reconstruction based on the *COI* sequences. There were a total of 618 positions in the final dataset for 28S D2 sequences and 628 positions for *COI* sequences in all phylogenetic reconstructions. Sequences of all haplotypes are deposited in GenBank (accession numbers are given in Additional file 1).

### Geometric morphometric analyses

**Figure 7:**
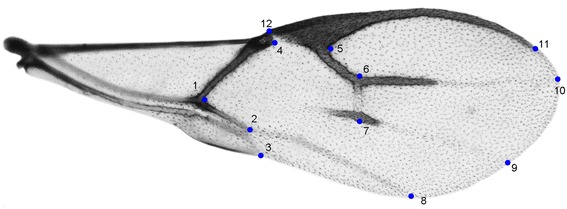
***Lysiphlebus*** **forewing with 12 selected landmarks.**

The geometric morphometric analyses were carried out on the right forewing. Microscopic slides of the wings were prepared in Berlese medium [45] and photographed using a Leica System Microscope DM2500 with a Leica DFC490 Digital Camera. Twelve landmarks were positioned using the TPSDIG2 software package [46] (Figure 7). The landmarks 1–5 and 12 define the proximal part of the forewing; the distal part of the wing is defined by the landmarks 6–11. The landmarks 8, 9 and 10 are projections of the three veins on the wing edge. Stigma and radial abscissa 1 (R1) were defined by the landmarks 11 and 12 (12 is the very apex of the stigma, 11 is the end of R1 vein); the landmarks 5 and 6 mark the first sector of the radial vein; and the vein between the landmarks 6 and 7 is defined as 2SR. The terminology used in this paper regarding the forewing venation of the aphidiines follows Sharkey & Wharton [47].

We applied the Generalised Procrustes Analysis (GPA) to obtain the matrix of the shape coordinates (also known as Procrustes coordinates) from which the differences due to position, scale and orientation have been removed [48,49]. The general size was computed as the centroid size (CS), which reflects the amount of dispersion around the centroid of the landmark configuration. The shape variables (Procrustes coordinates) were obtained using the MorphoJ software [50].

#### Divergence in wing size and shape

The divergence in wing size among *a priori*-defined groups based on phylogenetic clades, morphs or reproductive mode was analysed with Analyses of Variance (ANOVA). Post-hoc tests (Tukey’s Studentised Range) were used to test for differences between specific groups. The divergence of wing shape among groups was analysed with a Multivariate Analysis of Variance (MANOVA) using the PROC GLM procedure in SAS [51]. To visualise the patterns of variation in wing shape, a Principle Components Analysis (PCA) on the covariance matrix of wing shape variables was carried out. We also mapped geometric morphometric data onto the molecular phylogeny and tested for a phylogenetic signal in wing shape. The generalised method of least squares [52,53] was used to find the values for the internal nodes of the phylogeny from the shape averages of the terminal taxa [53-55]. The phylogenetic signal in the shape data was tested by a permutation approach using the MorphoJ software [50], which simulated the null hypothesis of a complete absence of phylogenetic structure by randomly reassigning the phenotypic data to the terminal nodes [56].

To quantify the shape differences, Procrustes distances were calculated between each pair of analysed groups. The statistical significance of differences in the mean shape between groups was estimated using a permu tation test based on 10,000 iterations. The statistically significant differences after Bonferroni correction are presented.

Differences among the *a priori*-defined groups (clades, morphs, and reproductive modes) were explored with a Discriminant Function (DF) analysis. We report both original and cross validation percentages to better estimate the uncertainty in assigning individuals to groups based on wing shape [57]. To further explore variation among the sexual and asexual lineages, we performed separate PCA analysis of the covariance matrices of wing shape data for the species groups “*fabarum*” and “*testaceipes*”. To determine the degree to which wing shape variation was associated with size variation (allometry), we performed a multivariate regression of wing shape variables on log CS. The multivariate regression was performed for the “*fabarum*” and “*testaceipes*” species groups separately. To explore allometry-free shape data, we performed PCA of covariance matrices using residuals obtained from multivariate regression as size-free shape variables.

### Availability of supporting data

The data sets supporting the results of this article are available in the Dryad.org repository, doi:10.5061/dryad.9t0g2, http://datadryad.org/review?doi = doi:10.5061/dryad.9t0g2.

Nucleotide sequences are submitted to GenBank database under the accession numbers [GenBank: KP663427- KP663464].

## Additional files

Additional file 1:
**Additional table.** Sampling data for *Lysiphlebus* specimens used for molecular analyses. Data about locality, aphid and plant host, date of collection, mode of reproduction, haplotypes, as well as GenBank accession numbers are given for each specimen. Additional file 2:
**Maximum Likelihood phylogenetic tree of** ***Lysiphlebus*** **haplotypes obtained from nuclear second expansion segment of** ***28S rRNA*** **gene sequences.** Numbers above/below the branches represent the bootstrap values (only values above 50% are shown) in following order: Maximum Likelihood/Maximum Parsimony/Neighbour-Joining. Scale bar indicates substitutions per site. Species/morphs, number of specimens and mode of reproduction are indicated in parentheses next to haplotype: Lfa – *L. fabarum* morph, Lca – *L. cardui* morph*,* Lco *– L. confusus* morph*,* Lme – *L. melandricola*, Lhi *– L. hirticornis,* Lte *– L. testaceipes,* Lor *– L. orientalis*; A – thelytokous (asexual) mode of reproduction, S - arrhenotokous (sexual) mode of reproduction. For more haplotype information see Additional file 1.
