# Supplementary material for: Genetic and morphological variation in sexual and asexual parasitoids of the genus Lysiphlebus – an apparent link between wing shape and reproductive mode
Source: BMC Evol Biol. 2015 Feb 4;15:5. doi: 10.1186/s12862-015-0293-5 (PMC4332431; doi:10.1186/s12862-015-0293-5)
Supplement: Additional file 2: — Maximum Likelihood phylogenetic tree of Lysiphlebus haplotypes obtained from nuclear second expansion segment of 28S rRNA gene sequences. Numbers above/below the branches represent the bootstrap values (only values above 50% are shown) in following order: Maximum Likelihood/Maximum Parsimony/Neighbour-Joining. Scale bar indicates substitutions per site. Species/morphs, number of specimens and mode of reproduction are indicated in parentheses next to haplotype: Lfa – L. fabarum morph, Lca – L. cardui morph, Lco – L. confusus morph, Lme – L. melandricola, Lhi – L. hirticornis, Lte – L. testaceipes, Lor – L. orientalis; A – thelytokous (asexual) mode of reproduction, S - arrhenotokous (sexual) mode of reproduction. For more haplotype information see Additional file 1. [file 12862_2015_293_MOESM2_ESM.pdf]

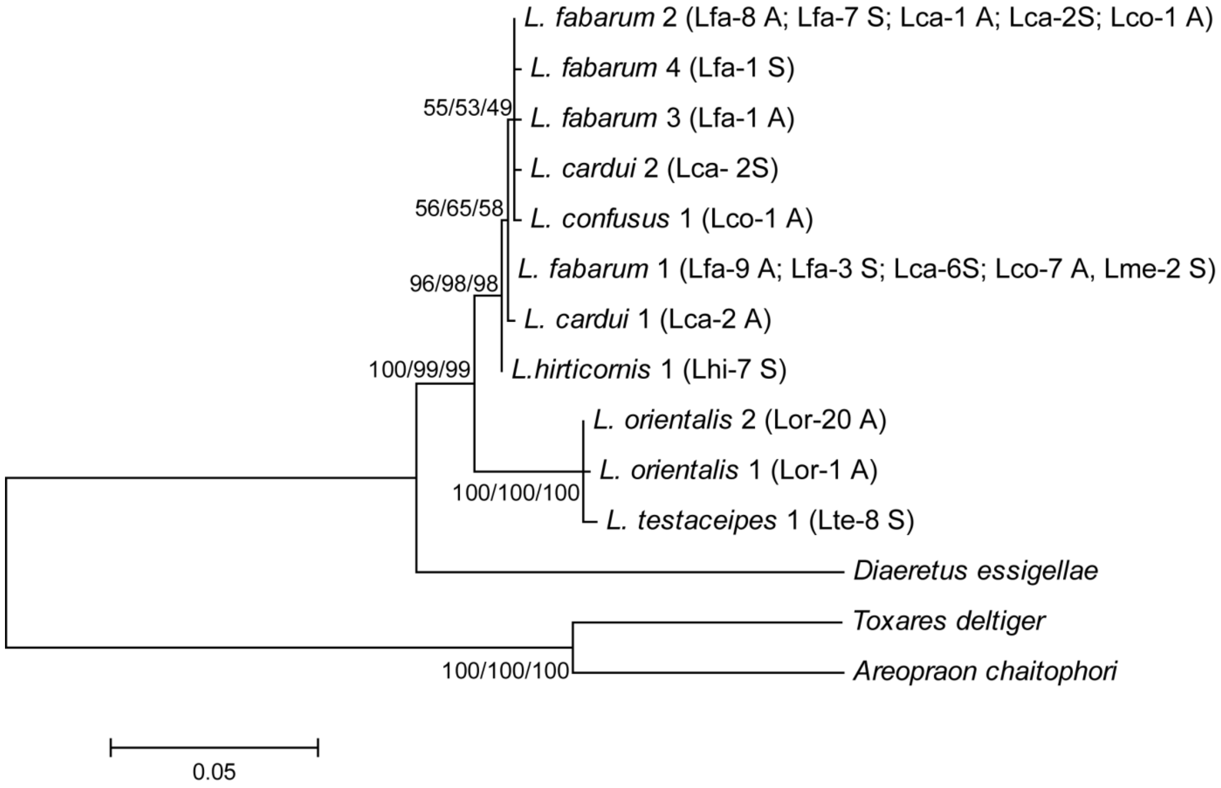

Additional Figure. Maximum Likelihood phylogenetic tree of *Lysiphlebus* haplotypes obtained from nuclear second expansion segment of 28S rRNA gene sequences. Numbers above/below the branches represent the bootstrap values (only values above 50% are shown) in following order Maximum Likelihood/ Maximum Parsimony/ Neighbor Joining. Scale bar indicates substitutions per site. Species/ morphotypes, number of specimens and mode of reproduction are indicated in parenthesis next to haplotype: Lfa – *L. fabarum* morphotype, Lca – *L. cardui* morphotype, Lco – *L. confusus* morphotype, Lme – *L. melandricola*, Lhi – *L. hirticornis*, Lte – *L. testaceipes*, Lor – *L. orientalis*; A – thelytokous (asexual) mode of reproduction, S - arrhenotokous (sexual) mode of reproduction. For more haplotype information see Additional file 1.
